# Cultivable Yeast Microbiota from the Marine Fish Species *Genypterus chilensis* and *Seriolella violacea*

**DOI:** 10.3390/jof7070515

**Published:** 2021-06-28

**Authors:** Benjamín Valderrama, José J. Ruiz, María Soledad Gutiérrez, Katherine Alveal, Mario Caruffo, Marcia Oliva, Héctor Flores, Alfonso Silva, Magaly Toro, Angélica Reyes-Jara, Paola Navarrete

**Affiliations:** 1 Laboratory of Microbiology and Probiotics, INTA, University of Chile, El Líbano 5524, Macul, Santiago 7830490, Chile; bpvalderrama@uc.cl (B.V.); ruiz.jose.90@gmail.com (J.J.R.); soleguti@uchile.cl (M.S.G.);mcaruffo@uchile.cl (M.C.); magaly.toro@inta.uchile.cl (M.T.); areyes@inta.uchile.cl (A.R.-J.); 2 Agencia Nacional de Investigación y Desarrollo (ANID)—Millennium Science Initiative Program—Millennium Nucleus in the Biology of the Intestinal Microbiota, Santiago 7830490, Chile; 3 Laboratorio de Cultivo de Peces, Departamento de Acuicultura, Facultad de Ciencias del Mar, Universidad Católica del Norte, Larrondo 1281, Coquimbo 1781421, Chile; kalvealz@hotmail.com (K.A.); moliva@ucn.cl (M.O.); hflores@ucn.cl (H.F.); asilva@ucn.cl (A.S.); 4 Escuela de Biotecnología, Facultad de Ciencias, Universidad Santo Tomás, Santiago 8370003, Chile; 5 Doctorado en Acuicultura, Programa Cooperativo Universidad de Chile, Universidad Católica del Norte, Pontificia Universidad Católica de Valparaíso, Valparaíso 2373223, Chile

**Keywords:** yeast, Rhodotorula mucilaginosa, Candida palmioleophila, Candida pseudorugosa, Cystobasidium slooffiae, Yamadazyma, Genypterus chilensis, Seriolella violacea, ITS region, hydrolytic enzymes

## Abstract

Because of its outstanding biological and industrial importance, many efforts have been made to characterize the mycobiota of new environments and their biochemical and biotechnological potentials. Gut mycobiota can be a source of novel yeasts with the potential to be used as probiotics or have industrial applications. In this work, we characterized two as-yet unexplored yeast communities from the intestinal content of the cultured marine Chilean fishes *Genypterus* *chilensis (G. chilensis)* and *Seriolella violacea* (*S.* *violacea*). Yeasts were isolated through culture, identified by sequencing their ITS region, and characterized their enzymatic profile with API^®^ZYM. *Rhodotorula mucilaginosa* was identified in both fish species. For the first time, *Candida palmioleophila*, *Candida pseudorugosa*, *Cystobasidium slooffiae,* and a member of the *Yamadazyma* genus were also identified and described as part of the normal fish gut–microbiota. Furthermore, the diverse enzymatic profile exhibited by some of these isolates suggests that it may be possible to develop novel applications for them, such as new probiotics and other biotechnological applications.

## 1. Introduction

Substantial efforts have been made to characterize the mycobiota of new environments and their biochemical potential, and some researchers have examined yeast communities living in the gut of diverse animals. As a result, some biotechnological applications of fish intestinal yeasts have been made [1], such as the industrial use of tannase-producer isolates [2]. Nevertheless, many gut–microbial communities and the yeasts living there remain underexplored; moreover, their biochemical potentials are still uncharacterized. It is widely known that gut microbial communities can influence various physiological features in the human host and fish [3,4,5]. Although the bacterial portion of these communities is constantly explored, their yeast counterparts’ role is far less investigated and, if so, is mainly remitted to their role in infectious diseases. For example, a report exists on the bacterial microbiota of *Genypterus* *chilensis* (*G. chilensis*) [6], whereas its yeasts have not been explored.

The International Scientific Association for Probiotics and Prebiotics (ISAPP) defines a probiotic as a “live microorganisms that, when administered in adequate amounts, confer a health benefit on the host” [7]. Previous studies showed that yeasts could exert probiotic effects by stimulating the host intestinal physiology [8] and immune system [9,10]. Moreover, fish’s immune response has been stimulated by either yeast or its isolated components mannan oligosaccharide and β-glucans [11]. Indeed, some authors already suggested some possible industrial applications for these yeasts due to their beneficial outputs. Although the probiotic effects of some yeasts are well documented, and yeasts have been identified in natural environments [3,12], there is still a lack of knowledge about potential uses of yeasts and the search for new gut-residents is still ongoing. Indeed, a fruitful way to shed light on novel applications for yeast is to expand the habitat to be analyzed.

In this work, we characterized two so far unexplored yeast communities isolated from the intestinal content of the fishes *G. chilensis* and *Seriolella violacea* (*S. violacea*). Yeast isolates were identified by sequencing their ITS regions and characterized by their enzymatic profile. As a result, four yeast species were identified: *Candida palmioleophila* (*C. palmioleophila*), *Candida pseudorugosa* (*C. pseudorugosa*), *Cystobasidium slooffiae* (*C. slooffiae*), and *Rhodotorula mucilaginosa* (*R. mucilaginosa*), which was also identified in both fish species. Additionally, other isolates were identified as members of the *Candida*, *Filobasidium*, *Diutina,* and *Yamadazyma* genera. Importantly, the last was described for the first time to be part of the gut–microbiota of fishes. Furthermore, the enzymatic profile exhibited by some isolates suggests that it is possible to develop applications as new probiotics, for example, to enhance the digestive capacities of fish.

## 2. Materials and Methods

### 2.1. Fish, Intestinal Collection and Ethics Statement

Healthy captive *S. violacea* adults (*n* = 38) of average weight 2.5–3.0 kg were collected from the Fish Culture Laboratory of the Universidad Católica del Norte. *G. chilensis* (*n* = 10) of similar characteristics were obtained from the fish culture center Colorado Chile S.A. Two procedures for intestinal sampling were applied depending on the possibility to sacrifice the fish: *S. violacea* were sacrificed with an overdose of anesthesia through fish immersion into a water solution containing 2-phenoxyethanol (4 mL L^−1^). The complete intestine of each fish was sampled in sterile conditions for further analysis. *G. chilensis* were anesthetized using ethyl-p-aminobenzoate (0.04 g L^−1^) for 5 min, and their intestinal contents were obtained with a sterile cannula. Intestinal samples were processed in the laboratory according to previously described protocols [13]. In brief, digesta and mucosa were homogenized with sterile phosphate-buffered saline (PBS) in a high-speed homogenizer for 5 min, and six ten-fold serial dilutions were made with sterile PBS. All fish experiments were performed according to approved protocols by the Ethics Committee for Animal Use of the Universidad Católica del Norte (Protocols Resol. 77/2017).

### 2.2. Isolation of Yeasts from Intestinal Samples by Culture Method

One hundred µL of each homogenized sample dilution were plated in duplicates on Yeast-extract Peptone Dextrose medium (YDP; 1% yeast extract; 2% peptone; 2% glucose, and 1.5% agar) supplemented with 0.05% chloramphenicol. Plates were incubated at 28 °C for up to 7 days. All morphologically different colonies from plates containing 1–100 colonies were re-isolated in YPD agar and microscopically confirmed as yeast by cell morphology.

### 2.3. Yeast DNA Extraction and Identification

DNA from each yeast isolate was extracted using the Wizard^®^ Genomic Kit (Promega, Madison, WI, USA) with a pre-incubation step of 1 h with lyticase (0.5 mg·mL^−1^) [13]. ITS amplicons were obtained with ITS1 (5′- TCCGTAGGTGAACCTGCGG-3′) [14] and NL4 (5′-GGTCCGTGTTTCAAGACGG-3′) primers [15]. PCR reactions were performed in 50 µL containing 0.5 µL of ITS1 and NL4 primers (both 20 µM); 25 µL of GoTaq^®^ G2 Green Master Mix 2X (Promega, Madison, WI, USA); 1.7 µL yeast DNA (100 ng µL ^−1^) and 22.3 µL of nuclease-free H_2_O. PCR consisted of an initial denaturation at 95 °C for 5 min, followed by 30 cycles comprising a denaturation at 94 °C for 30 s, annealing at 50 °C for 30 s and an extension at 72 °C for 1.5 min and 10 extra minutes at 72 °C. To identify yeast isolates, PCR products were purified and sequenced with ITS1 primers by Psomagen USA services.

### 2.4. Bioinformatic Workflow

Electropherograms of each DNA sequence were analyzed and edited with the Geneious v10 software [16]. Using BLAST [17] and NCBI nucleotide collection (nr/nt) database, all sequences were matched to a reference entry according to previous detailed works [18] and discussed standards [19,20]. Sequences considered for the analysis were those above the 90% coverage threshold. Identification at the species level was assigned when the identity percentage was at least 97%. Genus was confirmed when several species from the same genus, all with an identity score between 96 to 100%, showed the same BLAST top scores. Genus were also assigned when BLAST top scores showed identity scores between 90 to 96%, but several species belonged to the same genus, as previously recommended [18]. Additionally, for sequences identified at the species level, results were verified with Mycobank. Available online: http://www.mycobank.org (accessed on 15 December 2020). All selected sequences were aligned using the multiple sequences alignment (MSA) web tool Clustal Omega [21] with the default parameters. The remaining analysis were conducted in R v.4.0.2 [22], and the MSA output was imported into R with the “seqinr” package [23]. The “ape” package [24] was used for calculating distances between aligned reads using the Kimura’s-2-parameters distance method [25] and for constructing a phylogenetic tree. The phylogenetic tree was constructed for all the isolates and their closest relatives using the Neighbor-Joining method [26] with 1000-repetitions bootstrap [27]. The “ggtree” package was used to handle metadata and for visualization purposes [28,29,30]. All sequences obtained from yeast isolates were deposited in the GenBank database under the accession numbers MW567218 to MW567237.

### 2.5. Enzymatic Characterization of Yeasts

The API^®^ZYM test (Bio-Mérieux, Marcy l’Etoile, Auvergne-Rhône-Alpes, France) was performed according to the manufacturer’s instructions. The kit detects the activity of 19 enzymes, including lipases, proteases, glycosidases, and phosphohydrolases. Enzyme activity was recorded as positive or negative according to the reaction color intensity after incubating the strip with yeast for 5 min and then comparing it with the API-ZYM color reaction chart.

## 3. Results

### 3.1. Identification and Phylogeny of Isolated Yeasts

Out of the 48 fish in the study—38 *S. violacea* and 10 *G. chilensis—*we isolated yeast from five individuals of each species, representing 13.2% and 50.0% of prevalence, respectively.

**Figure 1:**
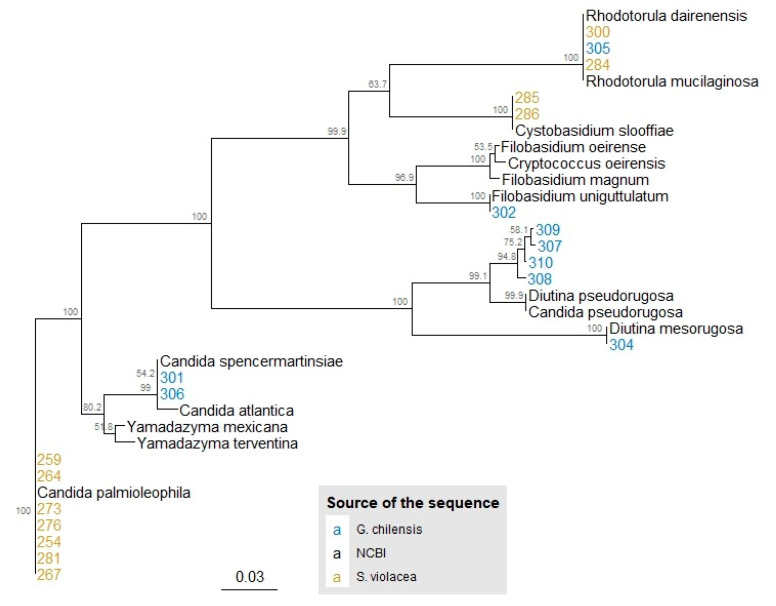
Phylogenetic tree of the 20 yeast isolated from *Seriolella violacea* and *Genypterus chilensis.* Phylogeny inferred with the Neighbor-Joining method and Kimura-2-parameters with 1000 bootstrap. Colored numbers represent the ID for one particular isolate. Isolates ID depicted in yellow were extracted from *S. violacea,* and those from *G. chilensis* are shown in blue. Sequences downloaded from NCBI (the closest relatives to the isolates in the study) are depicted in black.

Twenty phenotypically distinct colonies were selected; 11 from *S. violacea* and nine from *G. chilensis*. The ITS region was sequenced to identify each yeast isolate (Table 1). Of the total isolates retrieved from *S. violacea*, 64% (7/11) were classified within the Ascomycota division and 26% (4/11) within Basidiomycota. Additionally, 78% of recovered isolates from *G. chilensis* were Ascomycota and 22% Basidiomycota. *R. mucilaginosa* was identified in both fish species. While *C. palmioleophila*, *Rhodotorula dairenensis* (*R. dairenensis*), and *C. slooffiae* were detected only in *S. violacea*, *Candida* sp., *Filobasidium* sp., *Yamadazyma* sp., *Diutina mesorugosa* (*D. mesorugosa*), and *C. pseudorugosa* were recovered only from *G. chilensis*. A phylogenetic tree was constructed to show the evolutionary relationship between the closest relatives described in Table 1 and the sequences of the isolates in the current study (Figure 1).

### 3.2. Enzymatic Characterization of Yeasts

**Figure 2:**
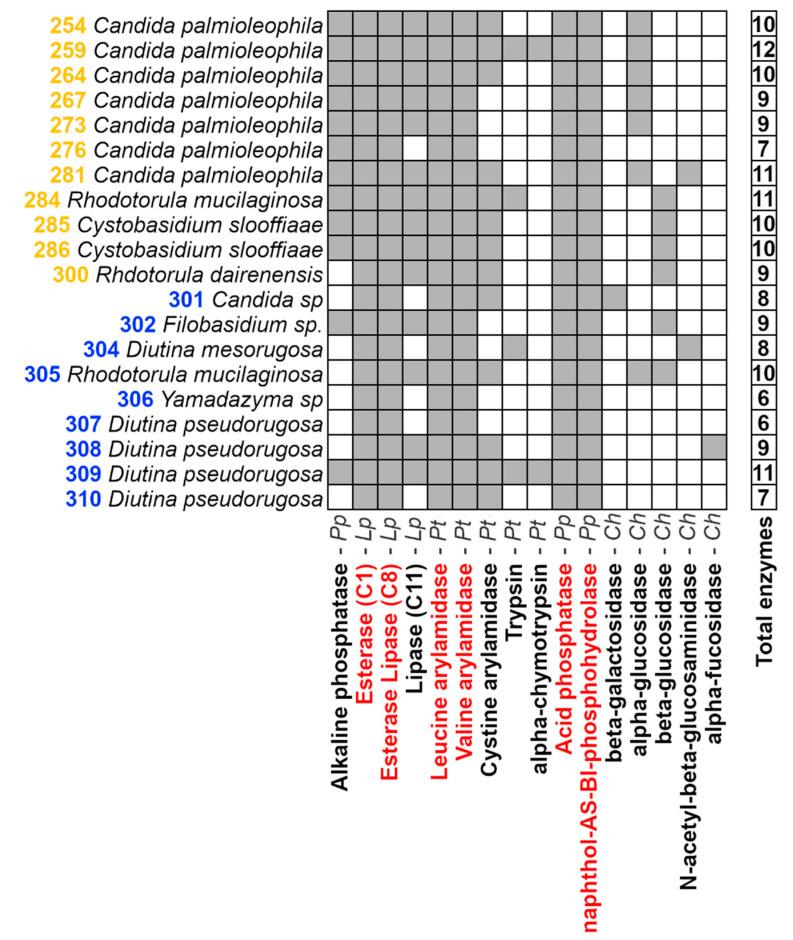
Hydrolytic enzymes detected (gray filled squares) in yeast isolates using API^®^ZYM Biomérieux. Isolates in yellow were recovered from *S. violacea* and those in blue from *G. chilensis*. The enzymes shared by all the samples are shown in red. Pp: Phosphatase, Lp: Lipase, Pt: Protease, and Ch: glycosidases.

An enzymatic characterization for the 20 yeast isolates was conducted with the API^®^ZYM (Bio-Mérieux, Marcy l’Etoile, Auvergne-Rhône-Alpes, France) test (Figure 2). The *in vitro* assay detected three phosphatases, three lipases, five proteases, and five glycosidases from the isolates. However, no reaction for neither α-galactosidase, β-glucuronidase, nor α-mannosidase was detected. Within the 16 enzymes detected, six of them were present in all of the isolates: esterase (C4), esterase-lipase (C8), leucine arylamidase, valine arylamidase, acid phosphatase, and naphthol-AS-BI-phosphohydrolase. Additionally, enzymes showing prevalences below 10% of the isolates were α-fucosidase and β-galactosidase (both detected in just one isolate) and N-acetyl-β-glucosaminidase and α-chymotrypsin (detected in two isolates each).

## 4. Discussions

In this study, we isolated yeasts from the gut microbiota of two cultivated fish species. Our results show a yeast prevalence of 13.2% and 50.0% in *S. violacea* and *G. chilensis*, respectively, which is lower than previous results obtained in other carnivorous Chilean fishes with a prevalence of at least 75% [13]. In the same study, *R. mucilaginosa* and *Debaryomyces hansenii* were identified as part of the common yeast species of the carnivorous fish species analyzed (reared and wild salmonids, yellowtail (*Seriola lalandi*), and croaker (*Cilus gilberti*)) [13]. Simultaneously, in our analysis, only *R. mucilaginosa* was found to be present in both fish species. This could be explained because the study of Raggi et al., 2014 included a more comprehensive analysis to describe the fish yeast gut microbiota, including a molecular method (PCR-TTGE) and culture. Interestingly, this is the first study reporting *C. palmioleophila*, *C. pseudorugosa*, *C. slooffiae*, and *Yamadazyma* sp. as part of the gut mycobiota of healthy fishes.

The yeast identification process here conducted was straightforward for almost every isolate with the following three considerations: (І) the sequences 307, 308, 309, and 310 matched with the same entries annotated in the database as either *C. pseudorugosa* or *D. pseudorugosa*. Despite this, within the metadata of the entry associated with *C. pseudorugosa* it is specified that the source organism is, in fact, *D. pseudorugosa*. Moreover, in the reference article, the authors defined the *Diutina* genus by reassigning some species from the *Candida* genus [31], so *D. pseudorugosa* seems to be the correct taxa identified. (ІІ) For sequence 302, the BLAST result of 94% of identity allowed its identification just at the genus level, which was also confirmed by the phylogenetic tree, which includes sequences of *Filobasidium oeirense* (BLAST’s best match), *Cryptococcus oeirensis*—which is a homotypic synonym of the former [32,33], *Filobasidium magnum* and *Filobasidium uniguttulatum* (tree’s closest sequence supported for high bootstrap values). Other marker genes would be necessary for identification that solves the discrepancy between BLAST’s best match and phylogeny’s closest sequence. (ІІІ) Similarly, isolate 306 was assigned to the *Yamadazima* genus based on the 94% identity obtained in the BLAST analysis. However, in the tree, the isolate was spotted closer to sequences of the *Candida* genus and isolate 301 (also assigned to *Candida*). It is worth noting that, in the study which describes *Yamadazyma terventina* [34] (BLAST’s best match of 306 isolates), it is showed that their closest relatives are some members of the *Yamadazyma* as well as *Candida* genera; both genera belong to the *Saccharomycetaceae* family. Considering the already mentioned, again, for better yeast identification, other marker genes would be necessary.

Although yeast identification by comparing ITS sequences with BLAST is a regular procedure, discussions and warnings have been made about its taxonomical rather than technical limitations [18,35]. Indeed, some concerns regarding the suitability of ITS sequences deposited in the GenBank for taxonomical identification include (but are not limited to) that about 20–27% have presented improper, insufficient, or incorrect taxonomic identification, 14% of the sequences data contained IUPAC DNA ambiguities, 82% had no explicit reference to a voucher specimen, and at least 42% the sequences have insufficient (and in some cases complete lack of) updates of its metadata [35,36]. Nevertheless, previous suggestions to enhance reliability highlighting the importance of the thresholds used for taxonomic-level assignation and cross-check with other databases [18,19] have been followed in this work. Additionally, it is true that sequencing of other marker genes, such as genes RPB1 and RPB2 encoding two RNA-polymerase subunits or beta-tubulin (tub2/BenA) [19], can improve identification and provide a better resolution at the species level for those sequences only assigned at the genus level.

We have previously characterized the enzymatic profile of yeast isolated from other marine fish gut using the API^®^ZYM system (Bio-Mérieux, Marcy l’Etoile, Auvergne-Rhône-Alpes, France) [13]. Although with some differences, our previous results are coherent with this study. Enzymes detected in all our isolates-esterase (C4), esterase-lipase (C8), leucine arylamidase, valine arylamidase, acid phosphatase, and naphthol AS-BI-phosphohydrolase- were detected in at least 75% of the isolates in our previous study [13]. This suggests the relevance of these enzymes either in the metabolism of yeast in this habitat or in the physiology of these carnivorous fishes. Indeed, it has been suggested that the microbiota composition and its metabolic capabilities respond to the host’s diet [37]. Accordingly, the enzymatic profile shown by these yeasts was comprised of proteases, valuable for carnivorous fishes (such as *S. violacea* and *G. chilensis*), and other hydrolytic enzymes such as lipases, glycosidases, and phosphatases. The detection of all these enzymes is coherent with the substrates found in the feed consumed by these fishes, such as highly unsaturated fatty acids, hydrolyzed marine proteins, phospholipids, and algae [38,39]. It is worth highlighting that the enzymatic characterization conducted in this study was performed in in vitro conditions. In consequence, other enzymes could be active when the yeasts grow in vivo in the fish.

Previous studies suggest that dietary supplementation with exogenous hydrolytic enzymes can enhance the digestive ability of fish larvae by compensating for low levels of host digestive enzymes and improving its nourishment [40]. In this context, yeasts can contribute to gut digestion by providing hydrolytic enzymes [41] or polyamines, which stimulate the expression and activities of brush border enzymes in intestinal cells [42,43].

It has been suggested that autochthonous yeasts isolated from the guts of closely related fishes are more successful in colonizing the digestive tract and exert more beneficial outputs to the host than non-autochthonous species [44,45]. Consequently, some of our yeast isolates could be tested for their probiotic potential in *S. violacea* and *G. chilensis,* which are native species that form part of the aquaculture diversification program in Chile. Specifically, it would be valuable to determine the effect of these yeast isolates on larvae survival, which is a critical bottleneck in producing these new fish species. This type of probiotics could positively impact the nourishment status of these local fishes and, therefore, have significant benefits in this food-production sector.

Additionally, previous studies have highlighted potential biotechnological applications for some of the yeast species identified here. For instance, a similar strain of *C. palmioleophila* has been recognized for its ability to remove oil and grease from water polluted with wastewater from palm oil refineries [46]. Likewise, *R. mucilaginosa* can use single-ringed aromatic hydrocarbons as a carbon source, and, therefore, its use has been proposed for environmental bioremediation [47]. *R. dairenensis* and other members of the genus are known as fructooligosaccharide producers, so they have been proposed as substitutes for the current production methods in the sweeteners industry [48]. Another yeast species identified with a described biotechnological application is *C. mesorugosa*, capable of producing indole-3-acetic acid, a growth-promoter agent in plants [49].

In the light of the previous examples, the importance of studying microbial communities in new environments becomes evident. By doing so, we can expand our knowledge of the profound impacts of the microbes in their environments, developing diverse industrial and biotechnological applications, or identify new sources of microorganisms with already known potentialities. Future studies will be performed to evaluate the probiotic or industrial potential of these isolates.

## Figures and Tables

**Table 1:** Identification of the yeast isolated from *Seriolella violacea* and *Genypterus chilensis.*

Fish Species	Fish Specimen	Isolate ID	Length of the Sequence (bp)	Closest Relative (NCBI Access Code of the Best Match)	Identity (%)	Query Cover (%)
*S. violacea*	1	254	960	*Candida palmioleophila* (KJ705005.1) *^a^*	99.8	100
	1	276	990	*Candida palmioleophila* (KJ705005.1) *^a^*	99.7	100
	1	273	972	*Candida palmioleophila* (KJ705005.1) *^a^*	99.5	100
	1	264	1029	*Candida palmioleophila* (KJ705005.1) *^a^*	99.8	100
	1	281	925	*Candida palmioleophila* (KJ705005.1) *^a^*	99.9	100
	1	267	1028	*Candida palmioleophila* (KJ705005.1) *^a^*	99.3	100
	1	259	1037	*Candida palmioleophila* (KJ705005.1) *^a^*	99.9	100
*S. violacea*	2	284	979	*Rhodotorula mucilaginosa* (MN006818.1*) ^b^*	99.7	99
*S. violacea*	3	300	976	*Rhodotorula dairenensis* (AB026010.2) *^b^*	99.6	100
*S. violacea*	4	285	1011	*Cystobasidium slooffiae* (AB025994.2) *^b^*	99.8	99
*S. violacea*	5	286	799	*Cystobasidium slooffiae* (AB025994.2) *^b^*	99.6	100
*G. chilensis*	1	301	626	*Candida* sp. (KP794187.1 and KY101952.1) *^a^*	99.3 99.3	94 94
	1	302	1065	*Filobasidium* sp. (KX067801.1) *^b^*	94.2	97
*G. chilensis*	2	304	841	*Diutina mesorugosa* (KY464166.1) *^a^*	99.9	100
*G. chilensis*	3	305	1018	*Rhodotorula mucilaginosa* (MN006818.1) *^b^*	99.8	99
*G. chilensis*	4	306	919	*Yamadazyma* sp. (JQ247716.1) *^a^*	94.3	100
*G. chilensis*	5	307	821	*Candida pseudorugosa* (KT336718.1) *^a^* *Diutina pseudorugosa* (MK394157.1) *^a^*	97.7 97.7	99 99
	5	308	828	*Candida pseudorugosa* (KT336718.1) *^a^* *Diutina pseudorugosa* (MK394157.1) *^a^*	98.6 98.6	96 96
	5	309	849	*Candida pseudorugosa* (KT336718.1) *^a^* *Diutina pseudorugosa* (MK394157.1) *^a^*	97.0 97.0	100 100
	5	310	803	*Candida pseudorugosa* (KT336718.1) *^a^* *Diutina pseudorugosa* (MK394157.1) *^a^*	98.1 98.1	100 100

The superscript letters “*^a^*” or “*^b^*” indicate whether a sequence corresponds to members of the divisions Ascomycota (a) or Basidiomycota (b).

## Data Availability

All the sequences obtained from the yeast strains were deposited in the GenBank database under the following accession number: MW567218 to MW567237.
